# Disentangling Effects of Input Frequency and Morphophonological Complexity on Children's Acquisition of Verb Inflection: An Elicited Production Study of Japanese

**DOI:** 10.1111/cogs.12554

**Published:** 2017-10-10

**Authors:** Tomoko Tatsumi, Ben Ambridge, Julian M. Pine

**Affiliations:** ^1^ Institute of Psychology University of Liverpool; ^2^ University of Liverpool and ESRC International Centre for Language and Communicative Development (LuCiD)

**Keywords:** Inflection, Morphology, Frequency, Complexity, Acquisition, Japanese

## Abstract

This study aims to disentangle the often‐confounded effects of input frequency and morphophonological complexity in the acquisition of inflection, by focusing on simple and complex verb forms in Japanese. Study 1 tested 28 children aged 3;3–4;3 on stative (complex) and simple past forms, and Study 2 tested 30 children aged 3;5–5;3 on completive (complex) and simple past forms, with both studies using a production priming paradigm. Mixed effects models for children's responses were built to test the prediction that children's verb use is explained by the relative bias in input frequency between the two inflectional forms. Although Study 1 did not show a significant effect of input bias (apparently due to problems with item selection), Study 2, which corrected for this problem, yielded the predicted relationship. These findings suggest that input frequency effects, at the level of different inflectional forms of the same verb stem, hold even after controlling for morphophonological complexity.

## 1 Introduction

Input frequency has been shown to be a key factor in explaining the pattern of children's language development. Indeed, it is almost certainly the factor that has received the greatest degree of empirical attention and support. Effects of input frequency have been observed in many different domains, including phonology, vocabulary, simple and complex syntax, and—the focus of the present article—inflectional morphology (see Ambridge, Rowland, Theakston, & Kidd, 2015; Ellis, 2002; for reviews). However, this factor is confounded with various other factors (e.g., Lieven, 2010; Tomasello & Stahl, 2004), most notably, at least in the domain of inflectional morphology, morphophonological complexity. Whether one is counting phonemes, morphemes, syllables, or objective duration, high‐frequency forms (e.g., *walk*) tend to be shorter and simpler than lower frequency equivalents (e.g., *walks, walking, ambulates*). Thus, virtually all previous findings of (apparent) frequency effects at the level of inflectional morphology could, in principle, be effects of morphophonological complexity in disguise. The goal of this study is to disentangle these two factors, by focusing on Japanese—a language whose system of agglutinative morphology allows for frequency and complexity to be dissociated at the level of individual verb stems.

### 1.1 Effects of frequency in child language

Input‐based accounts of language acquisition claim that patterns in children's usage and error reflect distributional properties of the input language. Although input‐based accounts have been proposed from a variety of theoretical perspectives (see Ambridge et al., 2015; for a review), they sit most naturally with the constructivist view, under which children build their linguistic knowledge on the basis of their experience with language, and hence are predicted to show frequency effects at all levels (e.g., Bybee, 2006; Ellis, 2002; Tomasello, 2003). Indeed, many studies have found a relationship between children's use of linguistic forms and the frequency with which these forms occur in the input language (e.g., Goodman, Dale, & Li, 2008; Naigles & Hoff‐Ginsberg, 1998; Smiley & Huttenlocher, 1995; Theakston, Lieven, Pine, & Rowland, 2002, 2004; Zamuner, Gerken, & Hammond, 2005).

The domain of inflectional morphology is particularly well suited to the investigation of frequency effects, because it is possible to look for relative frequency effects at the levels of individual verb (or noun) stems. It is generally agreed that because, in this domain, frequency effects arise as a result of probabilistic competition between semantically very similar forms (e.g., *play* vs. *plays*) it is the *relative* rather than *absolute* frequency of each form (most simply captured as a simple proportion or ratio) that is the appropriate input‐frequency measure (see Ambridge et al., 2015: 247 for discussion). For example, Räsänen, Ambridge, and Pine (2014) investigated children's errors of using bare/non‐finite forms instead of finite forms in English (e.g., *He play for *He plays*) and found that the by‐verb error rate in an elicited production study was explained by the proportional frequency of these verbs in bare versus 3sg *‐s* form in child‐directed speech. For example, *fit* occurred relatively often in 3sg form in Räsänen et al.'s input sample, and it was never produced incorrectly in the experiment, whereas *find* never occurred in 3sg form in the input sample and was produced incorrectly 50% of the time. Similarly, in a study of zero‐marking errors for English noun plural marking, Matthews and Theakston (2006) found an effect of relative input frequency such that, for example, children often produced errors such as *two mouse (for *two mice*) but rarely produced errors such as *two tooth (for *two teeth*), because the ratio of *mouse:mice* (roughly 7:1) is much greater than the ratio of *tooth:teeth* (roughly 1:6).

Importantly, particularly with regard to the present study, effects of proportional input frequency are observed not only for error rates, but also for patterns of (correct) usage. For instance, Tatsumi and Pine (2016) showed that, in Japanese, the proportional frequency of past‐inflected forms varies greatly across verbs in a child‐directed speech sample, and that this frequency measure predicted children's usage of the inflection. Specifically, the proportional frequency of past tense forms versus all other inflectional forms in each child's data correlated highly with that in his/her caregiver's child‐directed speech. Moreover, this result held even after controlling for the general semantic‐distributional properties of Japanese child‐directed speech by partialling out the proportional frequency of past tense forms averaged across the speech of the other caregivers in the sample.

### 1.2 Frequency and morphophonological complexity in verb inflection

On the face of it, the fact that by‐verb variation in the proportional frequency of different inflectional forms predicts both children's error rates and patterns of correct usage would seem to constitute powerful support for an account under which linguistic knowledge builds up as a result of input‐based learning. On closer inspection, however, this evidence is less than compelling because, in many of these cases, frequency is confounded with morphophonological complexity. This point is perhaps most clearly illustrated by studies of children's “defaulting” errors, so called because children are claimed to default to a particular form in the inflectional paradigm when they have difficulty retrieving the target form. For example, in a dense corpus study, Aguado‐Orea and Pine (2015) found that the majority of verb‐agreement errors produced by two Spanish‐speaking children involved the use of the third‐person singular (3sg) forms in non‐third‐person singular contexts (e.g., *Tú [2sg] *come* [3sg], for *Tú* [2sg] *comes* [2sg]. “you eat”). While Aguado‐Orea and Pine (2015) characterized these errors in terms of children's defaulting to the form of each verb with the highest input frequency (i.e., 3sg), at least two other defaulting‐based explanations are possible. First, 3sg could constitute a morpho‐syntactic default; a form that can be used even when its agreement features (third person, singular) are not licensed by the subject (e.g., Radford & Ploennig‐Pacheco, 1995). Second, as Aguado‐Orea and Pine (2015) themselves note, Spanish 3sg forms are not only the most frequent, but also the most phonologically prototypical and simple forms in the paradigm (e.g., *com**e*** vs. *com**es/emos/éis/en***), whether measured in phonemes, morphemes or objective duration. (In the present article, we abstract away from the question of how complexity is best measured, and use “morphophonologically complex” as a catch‐all term for forms that are longer on all three of these dimensions). Indeed, as the example of *com**e** for *com**es*** shows, the form to which children are defaulting is often—phonologically speaking—the target form with one or more phonemes omitted.

A similar situation holds for Finnish‐speaking children's person‐number agreement errors. Children sometimes misuse both the 3sg form (as in Spanish) and the 2sg imperative form in contexts in which these forms are ungrammatical (Räsänen, Ambridge, & Pine, 2016). Again, these forms are not only highly frequent forms but also morphophonologically simple forms. For example, the 2sg imperative form bears no overt morphological marking, and it is phonologically indistinguishable from the stem form (Laalo, 2003).

Consequently, it is not possible, for any of these previous studies, to determine whether apparent frequency effects are in fact caused by a confound with morphophonological complexity, underlining the need to distinguish these often‐confounded factors. One possible way to do so is to consider languages that show by‐verb variation in the frequency of morphophonologically simple and complex forms, such that the more complex form is less frequent than the corresponding simple form for some verbs, but *more* frequent than the corresponding simple form for others. The present study focusses on Japanese, which has this property for (at least) two classes of complex form: statives (Study 1) and completives (Study 2).

### 1.3 Morphological characteristics of Japanese verb inflection

Japanese has a relatively rich system of verb inflection in which a number of distinctions, including tense, aspect, voice, polarity, and politeness, are expressed by means of suffixation on verb stems. It is worth emphasizing that the notion of inflection in Japanese is different from “Latin‐type” fusional inflection. As Shibatani (1990: 221) states, inflectional endings “are fairly clearly segmentable, and the segmented endings (or suffixes) are correlated with inflectional categories in a one‐to‐one fashion.” The simplest verb form consists of a verb stem with a tense‐marking suffix (non‐past or past), as in *tabe‐ru* (eat‐NONPAST) and *tabe‐ta* (eat‐PAST). Because Japanese verb morphology is agglutinative, more suffixes can be attached to these simple forms, in each case between the stem and the tense‐marking suffix. In the present article, we focus on two aspectual suffixes: the stative and the completive marker.

The stative inflection^1^ is marked with the suffix ‐*te*, as in *tabe‐te‐ru* (eat‐STATIVE‐NONPAST), “be eating” and *tabe‐te‐ta* (eat‐STATIVE‐PAST), “was/were eating.” The meaning of this aspectual marker is usually described as stative or progressive (this paper uses the label “stative” for convenience), depending on the meaning of the verb. A stative interpretation is most natural when this suffix is combined with verbs that have the semantic feature of completion or achievement such as *tsuk*‐ “arrive,” as in *tsui‐te‐ru* “be arrived” (i.e., to be present). A progressive interpretation is most natural when this suffix is combined with action verbs, as for the above example of *tabe‐te‐ta*, “was/were eating” (e.g., *I was eating, when suddenly the phone rang*).

The completive inflection^2^ is marked with the suffix –*chaw* (which changes to *–chat* when followed by the PAST marker), as in *tabe‐chat‐ta* (eat‐COMPLETIVE‐PAST). Crosslinguistically, the completive form marks an action that has been done “thoroughly and to completion” (Bybee, Perkins, & Pagliuca, 1994:318), though in Japanese it often has an additional implication of negativity or unexpectedness (e.g., *I was on a diet but ate the whole meal*).

In terms of frequency of use, although complex forms generally outnumber simple forms in terms of types (i.e., different inflectional forms of the same verb stem), simple forms are generally more frequent in terms of tokens (in line with a language‐general distributional pattern; e.g., Anglin, Miller, & Wakefield, 1993; Zipf, 1935). For example, in the corpus counts used in the present Study 1, simple past forms outnumber stative past forms by almost a factor of 10 (7,545:791). This is not surprising, given the well‐known tendency to find a strong negative correlation between frequency and complexity, whereby frequently‐used forms become reduced and simplified. However, despite this general pattern, for some verbs, certain complex forms (including the stative and completive forms investigated in the present study) outnumber their corresponding simple forms. It is this feature of Japanese that makes it ideal for dissociating the roles of token frequency and morphophonological complexity.

### 1.4 Previous studies of frequency and morphophonological complexity in Japanese

The findings of a number of previous studies of Japanese verb inflection suggest that both (a) high input frequency and (b) morphological simplicity aid acquisition (to our knowledge, none have attempted to dissociate these factors). But at the same time, some studies have found that certain complex forms appear early and/or are used often by children.

Differences in the emergence and usage of inflected verb forms have been explained in terms of input frequency in many studies (e.g., Otomo, Miyata, & Shirai, 2015; Shirai, 1993). For example, as noted above, Tatsumi and Pine (2016) studied the distribution of inflectional forms in a naturalistic speech sample and demonstrated that children's usage of different inflectional forms of the same verb (e.g., *tabe‐ta* “eat‐PAST” vs. *tabe‐ru* “eat‐NONPAST”) correlates highly with that of their caregivers. In a follow‐up elicitation study, Tatsumi, Ambridge, and Pine (submitted) investigated the effect of input frequency in children's misuse of past‐tense forms in non‐past contexts and vice versa (e.g., *tabe‐ta* “eat‐PAST,” describing an action that is planned to take place *ashita*, “tomorrow”). The findings showed that children's error rate is explained by the by‐verb frequency distribution of these two tense forms in a sample of child‐directed speech. For example, children were most likely to use a past‐tense form in a non‐past context for a past‐biased verb such as *mitsuke* “find” for which the proportional frequency of past versus non‐past forms in the dataset was 0.96. Conversely, children were more likely to use a non‐past‐tense form in a past context for a non‐past‐biased verb such as *tsukaw* “use,” for which the proportional frequency of past versus non‐past forms was 0.11.

A higher degree of morphophonological complexity is generally considered to negatively affect learning. Many corpus‐based studies of verb inflection in child Japanese report that most of children's earliest forms are morphologically simple (e.g., Clancy, 1985; Otomo et al., 2015; Shirai, 1993, 1998; Shirai & Miyata, 2006). On the basis of these and similar observations, Iwatate (1981) and Takanashi (2009) proposed a learning process whereby children generally proceed from simple forms to complex forms by the addition of morphemes.

At the same time, however, some of these corpus studies show that certain complex forms are observed even in the very early stages, and they suggest that input frequency might be a particularly important factor for these complex forms (e.g., Clancy, 1985; Otomo et al., 2015). For example, Iwatate (1981) (though arguing that in general children learn simple forms earlier than complex forms) observed the production of complex forms like *tabechatta* (eat‐COMPLETIVE‐PAST) during the earliest observable stage (e.g., at 2;1).

Together, these previous studies suggest that both input frequency and morphophonological complexity are important factors in the acquisition of Japanese inflection, further highlighting the need for a study designed to pull apart these often‐confounded factors.

### 1.5 The present study

In summary, many previous studies of inflectional morphology (including studies of English, Finnish, Spanish and Japanese) have observed apparent effects of input frequency. However, since frequency is negatively correlated with morphophonological complexity, it remains possible that many apparent frequency effects are in fact complexity effects in disguise. The aim of the present study is therefore to dissociate these factors by focusing on Japanese; a language that shows by‐verb variation in the frequency of simple and complex forms, such that the more complex form is less frequent that the corresponding simple form for some verbs, but *more* frequent than the corresponding simple form for others.^3^ Specifically, we predict a positive correlation across verbs between the ratio of complex: simple forms in a representative input corpus and children's experimental production data. The two pairs of complex and simple forms are (**Study 1**) stative past (e.g., *tabe‐te‐ta* (eat‐STATIVE‐PAST) “was eating”) versus simple past (e.g., *tabe‐ta* (eat‐PAST) “ate”) and (**Study 2**) completive past (e.g., *tabe‐chat‐ta* (eat‐COMPLETIVE‐PAST) “have eaten”) versus simple past (e.g., *tabe‐ta* (eat‐PAST) “ate”). Thus, we predict that the proportion of stative versus simple forms produced by children in Study 1 will be higher for verbs like *wasure* “forget,” for which stative past forms outnumber simple past forms (17 tokens vs. 7 tokens in the child‐directed speech sample mentioned above) than verbs like *araw* “wash,” for which stative past forms are less frequent than simple past forms (1 tokens vs. 23 tokens). Similarly, we predict that the proportion of completive versus simple forms produced by children in Study 2 will be higher for verbs like *wasure* “forget,” for which completive past forms outnumber simple past forms (24 tokens vs. 5 tokens) than verbs like *moraw* “get,” for which completive past forms are less frequent than simple past forms (1 tokens vs. 21 tokens).

The reason for choosing these particular inflectional forms is that both statives and completives are relatively frequent in child (and child‐directed) speech. Importantly, because, in both cases, the difference in meaning between the complex and simple forms is mainly aspectual, it is straightforward to devise experimental settings in which the use of either form is natural.

## 2 Study 1: Simple past versus complex stative past

### 2.1 Method

#### 2.1.1 Participants

Twenty‐eight children (19 boys and 9 girls) aged 3;3–4;3 (*M* = 3;10), recruited from nurseries in Tokyo, participated in the experiment. All were native monolingual speakers of Japanese reported as showing no linguistic impairment.

#### 2.1.2 Design and materials

Twenty verbs were selected for use in the target pictures to be described by children: 10 biased toward simple past and 10 toward stative past forms in terms of input frequency. Frequency counts were taken from all combined child‐directed speech (both mothers and fathers) in the MiiPro corpus (Miyata & Nisisawa, 2009, 2010; Nisisawa & Miyata, 2009, 2010) in the CHILDES database (MacWhinney, 2000). For each verb, the token frequency of (a) the simple past form (e.g., *arat‐ta* “washed”) and (b) the stative past form (e.g., *arat‐te‐ta,* “was washing”) was obtained, using the FREQ function of the CLAN program (MacWhinney, 2000). In order to ensure that our experimental verbs spanned the full range of simple‐past‐biased and stative‐past‐biased verbs (see Table 1), verbs were selected, where possible, on the basis that they showed a bias that was significantly different from chance (i.e., from 50/50) by binomial test (*p *<* *.05). However, in order to obtain a sufficient number of verbs (bearing in mind their suitability for illustration in still pictures and familiarity to children), we also included 5 stative‐biased verbs and 2 simple‐biased verbs where the bias was not statistically significant. In addition to these 20 target verbs for use by children, 20 unbiased verbs (by binomial test) were selected for use by the experimenter (see Table 1).

**Table 1:** Target verbs for use by (a) children and (b) experimenter

	Verb	Meaning	Input Frequency (Simple Past)	Input Frequency (Stative Past)	Binomial Test	Verb Bias by Binomial Test	Directional Chi‐square Statistic (Log‐transformed)	Verb Bias by Chi‐square Statistic
(a) Children								
C1	Araw	Wash	23	1	<0.001	Simple	−0.58	Simple
C2	Fum	Step on	13	2	<0.001	Simple	0.23	Stative
C3	Hair	Enter	173	57	<0.001	Simple	4.18	Stative
C4	Hippar	Pull	11	1	<0.001	Simple	−0.02	Simple
C5	Taore	Fall down	11	2	0.002	Simple	0.42	Stative
C6	Nor	Ride	85	17	<0.001	Simple	1.97	Stative
C7	Tsukamae	Catch	26	1	<0.001	Simple	−0.72	Simple
C8	Mawar	Turn	6	3	0.090	Simple	1.94	Stative
C9	Nak	Cry	28	15	0.016	Simple	3.51	Stative
C10	Nom	Drink	10	5	0.059	Simple	2.39	Stative
C11	Wasure	Forget	7.0	17.0	0.011	Stative	4.67	Stative
C12	Shir	Know	0.0	23.0	<0.001	Stative	5.40	Stative
C13	Mot	Hold	11.0	13.0	0.271	Stative	4.04	Stative
C14	Mat	Wait	4.0	6.0	0.172	Stative	3.43	Stative
C15	Hashir	Run	0.0	3.0	<0.001	Stative	3.39	Stative
C16	Nokor	Remain	2.0	4.0	0.109	Stative	3.17	Stative
C17	Kabur	Put on (hat)	1.0	4.0	0.031	Stative	3.40	Stative
C18	Hak	Wear	1.0	4.0	0.031	Stative	3.40	Stative
C19	Waraw	Laugh	1.0	3.0	0.063	Stative	3.04	Stative
C20	Kakure	Hide	1.0	3.0	0.063	Stative	3.04	Stative
(b) Experimenter								
E1	Hik	Play	1.0	0.0	0.5	Unbiased	−0.10	Simple
E2	Sagas	Look for	3.0	2.0	0.5	Unbiased	1.86	Stative
E3	Odor	Dance	1.0	0.0	0.5	Unbiased	−0.10	Simple
E4	Oyog	Swim	2.0	0.0	0.25	Unbiased	−0.19	Simple
E5	Oki	Get up	2.0	2.0	0.688	Unbiased	2.16	Stative
E6	Asob	Play	13.0	16.0	0.77	Unbiased	4.27	Stative
E7	Narab	Line up	0.0	1.0	1	Unbiased	2.36	Stative
E8	Ur	Sell	2.0	1.0	0.5	Unbiased	1.09	Stative
E9	Tsukam	Grab	0.0	1.0	n.a	Unbiased	2.36	Stative
E10	Shaber	Chat	1	2.0	0.875	Unbiased	2.52	Stative
E11	Oshie	Teach	3.0	4.0	0.773	Unbiased	2.97	Stative
E12	Kuttsuke	Stick	1.0	1.0	0.75	Unbiased	1.57	Stative
E13	Aruk	Walk	5.0	2.0	0.227	Unbiased	1.38	Stative
E14	Fur	Rainfall	3.0	2.0	0.5	Unbiased	1.86	Stative
E15	Hos	Dry	1.0	2.0	0.875	Unbiased	2.52	Stative
E16	Mak	Wind	0.0	2.0	n.a	Unbiased	3.00	Stative
E17	Magar	Bend	2.0	1.0	0.5	Unbiased	1.09	Stative
E18	Yorokob	Be pleased	3.0	2.0	0.5	Unbiased	1.86	Stative
E19	Sak	Bloom	2.0	1.0	0.5	Unbiased	1.09	Stative
E20	Ker	Kick	3.0	1.0	0.313	Unbiased	0.75	Stative

However, after conducting this study (and Study 2), we realized that the simple proportion of simple versus stative past forms is not a true measure of verb bias. Because simple past forms outnumber stative past forms by a ratio of—in this dataset—around 10:1 (7,545 vs. 791), a verb that exhibits a more modest “preference” for simple forms—such as *nor*, “ride” (5:1, 85 vs. 17)—is actually biased *against* the simple form, and in favor of the stative form, at least in the context of all the verbs in the corpus. In other words, given that, on average, the Japanese speaker's communicative goals require the use of a stative past form around 8% of the time, a verb that occurs in the stative past form 20% of the time is displaying a rather considerable bias towards this form. To address this problem, as outlined in more detail below, we used a chi‐square statistic as a measure of verb bias. Note that the point of using this statistic is not to establish which verbs show a significant bias towards the simple or the stative form, but rather to provide a measure of verb bias that controls for the overall bias towards simple forms in the language as a whole.

#### 2.1.3 Procedure

Children were tested individually in a classroom or teachers' room. Each child completed a test session of approximately 10 min, together with a native Japanese‐speaking experimenter, in front of a laptop computer (Macbook Pro, 11 inch). Before the session, the experimenter told each child that they would play a bingo game together in which they would take turns to describe pictures, in order to win star cards to fill up a grid, with the first to fill all six squares declared the winner (e.g., Rowland, Chang, Ambridge, Pine, & Lieven, 2012). The experiment was presented using Processing (https://processing.org/).

For each trial, the computer presented a still picture depicting a scene, and an audio stimulus of the corresponding verb in a simple non‐past form (described to children as the “clue word”). The player (either the experimenter or the child) then described the picture. The experimenter, who always went first, always described the pictures using stative past forms in order to prime these forms for children (though simple past forms are also perfectly acceptable in this context). This priming for the stative past inflection was necessary in order to encourage children to use both stative and simple past forms. With no such priming children would have been likely to use exclusively simple past forms, because simple past inflection is generally far more frequent than stative past inflection, and because the relatively general and neutral meaning of simple past makes this form more natural when describing the pictures (it is not possible to create picture contexts that prefer stative to simple past forms). It is important to note that, because we primed only complex forms, we cannot conclude—if the results pattern in accordance with our predictions—that frequency *trumps* morphophonological complexity; without priming, children would probably have produced simple forms across the board. All that we can conclude—if the results pattern in accordance with our predictions—is that frequency effects are observed even after controlling for complexity; that is, that when we invite children to produce exclusively complex forms (by means of a priming task), their ability to do so depends on the frequency with which that verb appears in complex versus simple form.

For the child's turn, the experimenter, following the clue word, provided the target sentence except for the verb (always the final element of the sentence), to be supplied by the child. In order to highlight the past‐tense context (in which both stative past and simple past forms are totally acceptable), all sentences began with the temporal adverb *kinoo,* “yesterday.” The following example shows a set of experimenter and child turns.

**Table:** 

[Experimenter's turn]	
Computer:	*Hiku* (while showing a picture of a girl playing the guitar)
	play‐NON.PAST
	“play”
Experimenter:	*Kinoo Yuuchan wa gitaa o* *hii‐te‐ta*.
	yesterday Yuuchan TOPIC guitar ACC play‐STATIVE‐PAST
	“Yesterday Yuuchan was playing the guitar”

**Table:** 

[Child's turn]	
Computer:	*Arau* (while showing a picture of a girl washing her hands)
	wash‐NON.PAST
	“wash”
Experimenter:	*Kinoo Yuuchan wa te o*…
	yesterday Yuuchan TOPIC hand ACC
	“Yesterday Yuuchan… her hands”
Child:	*Arat‐te‐ta/Arat‐ta*
	wash‐STATIVE‐PAST/wash‐PAST
	“was washing/washed”

After each trial, the computer displayed a star or a cloud to indicate whether or not the player received a star card. This was independent of the response given, and followed a predetermined sequence that ensured that the child won each game. Each session consisted of two games, each consisting of 20 trials (10 turns for each player), such that every child completed a target trial for every verb. Children's responses were recorded using Audacity (http://www.audacityteam.org/) and a separate audio recorder for later transcription and coding (by the first author).

#### 2.1.4 Analyses

Children's responses were analysed on a trial‐by‐trial basis, using mixed effects models in R, coded as 1 if the target verb was used in (complex) stative past form and 0 in simple past form, with all other responses excluded (*N *=* *166). These excluded responses were 89 simple non‐past forms (reflecting use of the clue words), 10 other inflectional forms of the target verb such as stative non‐past forms, 34 responses using non‐target verbs, and 33 responses of other types such as nouns and adjectives (e.g., *hen* “strange”). The number of unscorable responses is not unexpected given the experimental design, in which children are free to produce any response, with no direct instructions, and is similar to that observed in comparable studies (e.g., Räsänen et al., 2014). Also excluded were a further 12 trials for which children produced no response and 1 trial for which the experimenter failed to provide the correct prime sentence, for a final total of 381 scorable responses.

Predictor variables were trial number (for investigating the possibility of incremental priming throughout each experimental session, given that the experimenter always produced stative past forms) and input bias (stative vs. simple past). In order to take into account the overall preponderance of simple past forms, this predictor was a chi‐square value (without Yates' correction) calculated from counts taken from the MiiPro corpus (Miyata & Nisisawa, 2009, 2010; Nisisawa & Miyata, 2009, 2010). This value (see the following formula and Table 2) represents the extent to which a verb's particular bias towards stative versus simple past forms (or vice‐versa) differs from the bias shown by all other verbs in the corpus. Because the chi‐square test is non‐directional, we set the sign to positive if the ratio of stative:simple past forms was greater for the target verb than for all other verbs, and otherwise to negative. The use of polarity (+/−) to indicate whether a verb is biased towards or against a particular morpheme or (more usually) construction is standard for this type of analysis (see, e.g., Gries, 2015, for discussion). Chi‐square values were natural‐log transformed (ln(1 + *n*)) prior to any polarity change.

$$\chi^{2}={\left(ad-bc\right)}^{2}\times \left(a+b+c+d\right)/\left(a+c\right)\left(c+d\right)\left(b+d\right)\left(a+b\right)$$

Linear mixed‐effects models were fit, using the lme4 package (Bates, Maechler, Bolker, & Walker, 2014) of the statistical program R (R Core team, 2015). In addition to the fixed effects above, the models included random intercepts for participant and verb, and as many random slopes as possible without causing convergence failure (Barr, Levy, Scheepers, & Tily, 2013). The model comparison (likelihood ratio test) method was used to determine the significance level of individual predictors (Baayen, Davidson, & Bates, 2008; Barr et al., 2013; Cohen‐Goldberg, 2012). This method involves sequentially adding predictors—here (1) trial number, (2) the chi‐square input‐bias predictor, and (3) the interaction—to an initial baseline model that includes random effects only, and comparing each pair of models by means of a chi‐square test (using the anova function of R).

**Table 2:** Contingency table for the chi‐square calculation

	Target Verb	All Other Verbs	Row Totals
Stative past form	a	b	a+b
Simple past form	c	d	c+d
Column totals	a+c	b+d	a+b+c+d

### 2.2 Results (Study 1)

**Figure 1:**
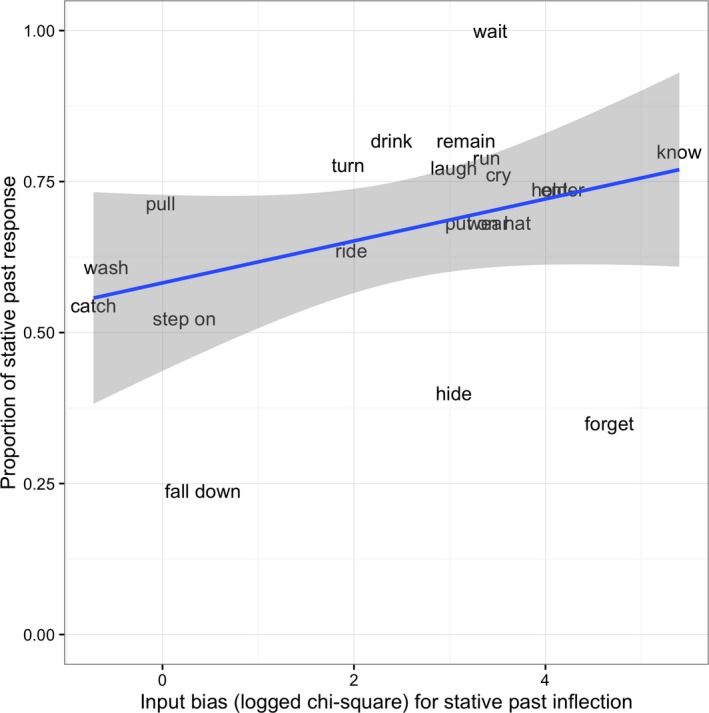
Proportion of children's stative versus simple past forms by input bias (data points are plotted with verb labels in English translation).

A binomial mixed effect model was fitted to children's responses (stative/simple past forms) with predictor variables of trial number and stative‐versus‐simple input bias (chi‐square measure; see Fig. 1), and the interaction. The final model that converged had by‐subject and by‐item intercepts, and a by‐subject slope for input bias. The model comparison procedure revealed no significant effect of trial number (β = −0.001, *SE* = 0.02, χ^2^ = 0.17, *p *=* *.68), indicating that priming did not build up over the course of the study. Nor did the addition of input bias (β = 0.36, *SE* = 0.29, χ^2^ = 1.58, *p *=* *.21) (see Fig. 1) or the interaction (β = 0.02, *SE* = 0.01, χ^2^ = 3.02, *p *=* *.08) significantly improve the model.^4^

### 2.3 Discussion (Study 1)

Although the trend was in the predicted direction, Study 1 found no evidence to support the hypothesis that children's relative by‐verb production of simple versus complex forms (here, simple‐ vs. stative‐past forms) reflects the frequency distribution of these forms in the input.

Of course, one possible reason for this failure to find the predicted effect is that there is no effect to find. Another is the influence of outliers: one (***mat*** **“wait”**) produced exclusively in stative‐past form (presumably because waiting tends to constitute a continuous activity) and three (***taore*** **“fall down,”** ***kakure*** **“hide,”** ***wasure*** **“forget”**) produced on over 50% of occasions in simple‐past form. Indeed, an analysis of the data with these four verbs removed showed a significant effect of input bias (β = 0.44, *SE* = 0.15, χ^2^ = 6.72 *p *=* *.01), with no significant effect of trial number (β = 0.01, *SE* = 0.02, χ^2^ = 0.02 *p *=* *.88) and no interaction (β = 0.01, *SE* = 0.01, χ^2^ = 1.25, *p *=* *.26). A third possible reason for the lack of an input effect is that children are not sensitive to the very subtle, mainly aspectual, semantic distinction between simple and stative forms. This possibility is supported by the lack of a priming effect, with children consistently producing stative forms at a rate of around 65% from the beginning of the experiment. A fourth and final possible reason for our failure to find an input effect lies with our choice of verbs. As shown in Fig. 1, although our experimental verbs were selected on the basis that 10 of them were simple‐biased and 10 complex‐biased in absolute terms, for all but three of these verbs (*catch, wash,* and *pull*), the chi‐square value is positive, indicating that the majority of verbs chosen as “simple‐past‐biased” were actually stative‐past‐biased in the context of all the verbs in the corpus. That is, although the remaining “simple‐past‐biased” verbs are more frequent in simple‐ than stative‐past in the input corpus, the extent of the bias is smaller than for verbs in the corpus in general—presumably because the design of the experiment forced us to choose “simple‐past‐biased” verbs that were nevertheless relatively natural in stative past form. This raises the possibility that our failure to find an effect of input bias reflects the failure to include verbs with a wide enough range of input biases to detect the effect.

In view of these possibilities, the absence of an input effect in Study 1 is difficult to interpret. We therefore conducted a second study designed to address the potential shortcomings of (1) outlier verbs, (2) insensitivity to the relevant semantic distinction, and (3) insufficient manipulation of the input‐based predictor. This second study focuses on the completive morpheme, which is both semantically more salient and also allows for the inclusion of verbs that are genuinely biased towards the simple form.

## 3 Study 2: Simple past versus complex completive past

### 3.1 Method

#### 3.1.1 Participants

Thirty children (12 boys and 18 girls) aged 3;5–5;3 (*M* = 4;2), recruited from nurseries in Tokyo, participated in the experiment. All were native monolingual speakers of Japanese reported as showing no linguistic impairment.

#### 3.1.2 Design and materials

As in Study 1, children took part in a sentence‐completion task that elicited simple past forms and (complex) completive past forms for 20 verbs (10 biased towards simple past and 10 towards completive past forms). These verbs were selected on the basis of the frequency distribution in the input, also taking into account their familiarity for children and the ease with which they could be illustrated. Frequency counts were taken from the same sources as for Study 1. For each verb, the token frequency of (a) the simple past form (e.g., *koware‐ta* “broke”) and (b) the completive past form (e.g., *koware‐chat‐ta* “have broken”) was obtained, using the FREQ function of the CLAN program (MacWhinney, 2000). In general, simple‐past‐biased verbs and completive‐past‐biased verbs (see Table 3) were selected on the basis that the relevant bias was significantly different from chance (i.e., to 0.5 simple past vs. stative past) by binomial test (*p *<* *.05). However, due to the fact that the instances of completive past verbs are not so abundant in the speech sample, we included 7 completive‐biased verbs for which the bias did not reach significance. In addition to these 20 target verbs for use by children, 20 unbiased verbs (by binomial test) were selected for use by the experimenter (see Table 3). However, after subsequently realizing the problems associated with raw proportions and the binomial statistic, we again calculated a chi‐square statistic as a measure of verb bias, in order to take into account the fact that simple past forms outnumber completive past forms (in this dataset by 7,545 to 1,047).

**Table 3:** Verbs for (a) children and (b) experimenter

	Verb	Meaning	Input Frequency (Simple Past)	Input Frequency (Completive Past)	Binomial Test	Verb Bias by Binomial Test	Directional Chi‐square Statistic (Log‐transformed)	Verb Bias by Chi‐square Statistic
(a) Children								
C1	Moraw	Get	21	1	<0.001	Simple	−0.79	Simple
C2	Tat	Stand	10	1	0.001	Simple	−0.09	Simple
C3	Kaw	Buy	49	3	<0.001	Simple	−1.10	Simple
C4	Mi	Look	57	2	<0.001	Simple	−1.67	Simple
C5	Mitsuke	Find	14	2	0.002	Simple	0.00	Unbiased
C6	Tsukur	Make	30	1	<0.001	Simple	−1.20	Simple
C7	Ire	Put in	17	2	<0.001	Simple	−0.05	Simple
C8	Tor	Take	28	3	<0.001	Simple	−0.17	Simple
C9	Kak	Write	37	3	<0.001	Simple	−0.60	Simple
C10	Nor	Ride	16	2	0.000	Simple	−0.02	Simple
C11	Nakunar	Disappear	15	31	0.013	Completive	4.89	Completive
C12	Otos	Drop	8	13	0.192	Completive	3.90	Completive
C13	Wasure	Forget	5	24	0.000	Completive	4.92	Completive
C14	Hazure	Come off	4	9	0.133	Completive	3.70	Completive
C15	Koware	Break	17	27	0.087	Completive	4.61	Completive
C16	Okkochi	Fall down	3	10	0.046	Completive	3.95	Completive
C17	Ware	Split	2	5	0.227	Completive	3.18	Completive
C18	Kire	Cut	1	5	0.109	Completive	3.38	Completive
C19	Nak	Cry	8	12	0.252	Completive	3.78	Completive
C20	Korob	Tumble	1	4	0.188	Completive	3.11	Completive
(b) Experimenter								
E1	Asob	Play	1	1	0.75	Unbiased	1.30	Completive
E2	Chirakas	Mess up	1	1	0.75	Unbiased	1.30	Completive
E3	Muk	Peel	0	1	0.5	Unbiased	2.11	Completive
E4	Hare	Swell	1	1	0.75	Unbiased	1.30	Completive
E5	Fue	Increase	0	1	0.5	Unbiased	2.11	Completive
E6	Mazar	Mix	1	1	0.75	Unbiased	1.30	Completive
E7	Tomar	Stop	5	4	0.5	Unbiased	2.28	Completive
E8	Nemur	Sleep	1	1	0.75	Unbiased	1.30	Completive
E9	Nokor	Be left	1	1	0.75	Unbiased	1.30	Completive
E10	Or	Bend	1	1	0.75	Unbiased	1.30	Completive
E11	Shime	Shut	1	1	0.75	Unbiased	1.30	Completive
E12	Sugi	Pass	1	1	0.75	Unbiased	1.30	Completive
E13	Tob	Fly	1	1	0.75	Unbiased	1.30	Completive
E14	Tsukaw	Use	1	1	0.75	Unbiased	1.30	Completive
E15	Yabur	Tear	0	1	0.5	Unbiased	2.11	Completive
E16	Yuzur	Cede	0	1	0.5	Unbiased	2.11	Completive
E17	Agar	Ascend	1	2	0.5	Unbiased	2.23	Completive
E18	Nug	Take off	1	2	0.5	Unbiased	2.23	Completive
E19	Okor	Get angry	1	2	0.5	Unbiased	2.23	Completive
E20	Watas	Give	1	2	0.5	Unbiased	2.23	Completive

#### 3.1.3 Procedure

The procedure was the same as for Study 1, the only difference being the use of the completive instead of the stative inflection in the experimenter's prime sentences.

#### 3.1.4 Analyses

Children's responses were dummy coded as 1 if the target verb was used in (complex) completive past form and 0 in simple past form, with all other responses excluded (*N* = 133). These excluded responses were 59 simple non‐past forms (reflecting use of clue words), 2 target verbs with non‐target inflections such as stative non‐past forms, 52 responses using non‐target verbs, and 23 responses of other types such as nouns and adjectives (e.g., *okashi* “snacks”). Also excluded were a further 5 trials for which children produced no response and 6 trials for which the experimenter failed to provide the correct prime sentence, for a final total of 455 scorable responses; giving an exclusion rate comparable with both Study 1 and previous studies. The data were analyzed in the same way as in Study 1, including the use of a chi‐square measure as the measure of input bias (here, completive vs. simple past).

### 3.2 Result and discussion (Study 2)

**Figure 2:**
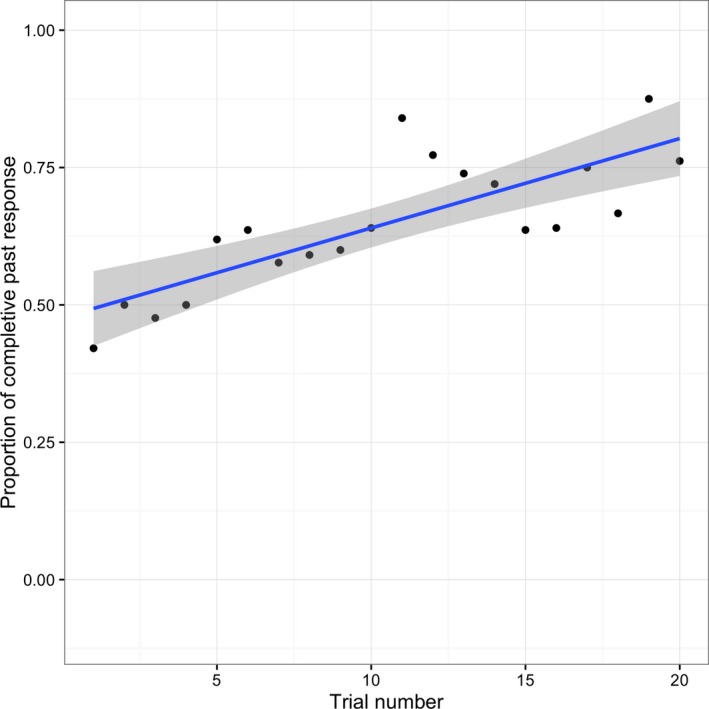
Proportion of children's completive versus simple past forms by trial number.

**Figure 3:**
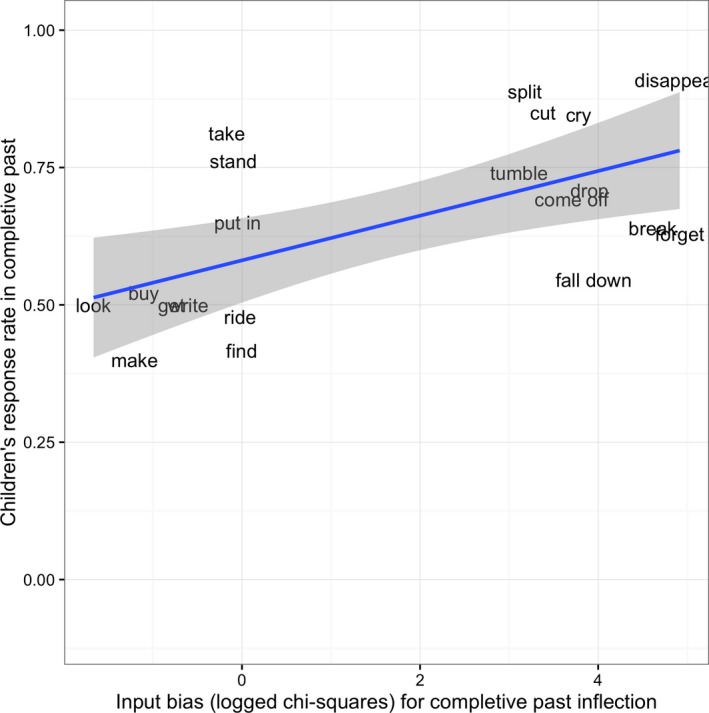
Proportion of children's completive versus simple past forms by input bias (data points are plotted with verb labels in English translation).

A binomial mixed effect model was fitted to children's responses (completive/simple past forms) with predictor variables of trial number (see Fig. 2), completive‐versus‐simple input bias (chi‐square measure; see Fig. 3), and the interaction between these variables. The final model had by‐subject random slopes for the input bias and by‐subject and by‐item random intercepts.

The model revealed a significant effect of trial number (β = 0.38, *SE* = 0.15, χ^2^ = 24.11, *p *<* *.001), indicating that children's use of completive past forms increased as a result of the incremental priming of these forms during the test session (see Fig. 2). Since trials were presented in random order, this effect cannot be responsible for any observed effect of verb bias. It does, however, have implications for the extent to which these findings can be generalized to children's everyday language use; a finding to which we return in the General Discussion.

Crucially, the addition of input bias was found to significantly increase model fit (β = 0.38, *SE* = 0.15, χ^2^ = 5.44, *p *=* *.02; see Fig. 3). Unlike in Study 1, all but one of the verbs selected as simple‐biased (*mitsuke*, “find”) did indeed show this bias, relative to the verbs in the corpus as a whole, on the chi‐square measure (i.e., the chi‐square measure has a negative value). The interaction between these factors did not reach significance (β = 0.02, *SE* = 3.00, χ^2^ = 5.44, *p *=* *.08) (though see note^4^).

In summary, the findings of Study 2 show that children's choice between simple and completive past forms reflects the probabilistic patterning of these whole inflected forms in the input language. More broadly, these findings suggest that children's knowledge of different inflectional forms reflects the relative strength of these forms in the input, and that the failure of Study 1 to find such an effect is a reflection of our failure to select verbs that were sufficiently biased towards simple‐past forms.

## 4 General discussion

The goal of the present study was to investigate whether effects of input frequency, as observed in a large number of previous studies, hold after controlling for a potentially confounding factor present in virtually all previous studies: morphophonological complexity. The present study focused on Japanese verb inflection, which constitutes a particularly suitable test case because morphophonologically complex forms outnumber simple forms for some verbs but are less frequent for others. Across both experiments, the prediction of a frequency‐based account is that the likelihood with which children produce simple versus complex forms for each verb reflects the relative frequency of the two forms in the input. Such an effect was found for simple versus complex *completive* past forms (Study 2), but not for simple versus complex *stative* past forms (Study 1).

Although, at first glance, the findings of Study 1 might appear to constitute evidence against a frequency‐based account, closer inspection of the set of verbs revealed (as well as a number of outliers) an insufficient number that were truly biased towards the simple past form. Although all verbs selected as “simple‐past‐biased” verbs were more frequent in simple‐ than stative‐past form in the input corpus, for all but three, the extent of the bias was smaller than for verbs in the corpus in general. This problem is difficult to avoid for statives, because the design of the experiment requires that all verbs selected be relatively natural in stative past form, hence ruling out verbs with a large simple‐past bias.

For Study 2, we therefore replaced statives with completives as the complex past form. The fact that the predicted effect of input frequency emerged in this study provides support not only for frequency‐based accounts, but also for the possibility that the failure to find such an effect in Study 1 was due to the methodological problems addressed by Study 2.

Nevertheless, two other differences between the two studies merit further consideration. First, for Study 2 (completives), the observed rates of simple versus complex form production across verbs were essentially as would be expected on the basis of the input sample, with no clear outliers. In contrast, for Study 1 (statives), one verb (*mat,* “wait”) and three verbs (*taore,* “fall down”; *kakure,* “hide”; *wasure,* “forget”) were produced in their complex stative form considerably more and less often respectively than would be expected on the basis of the input. (Indeed, recall that the input predictor reached statistical significance with these outliers removed). One possible explanation for these outliers is an effect of semantics. Waiting, by its very nature, tends to be a stative activity, while falling down and (possibly) hiding and forgetting tend to be more eventive or dynamic. Although we do not see any clear semantic tendency that could define the simple‐biased and complex‐biased verbs used in the study, these outlier verbs seem to highlight a point that is central to the present article: that any investigation of frequency effects must take great care to control for potentially confounding factors such as morphophonological complexity (which we controlled for largely successfully) and verb semantics (which we did not).

This brings us to the second important difference between the two studies. Although both stative (Study 1) and completive (Study 2) inflections mark aspectual distinctions and are roughly equivalent in terms of morphophonological properties (‐*te* vs. ‐*chat*), they differ with regard to the semantic distinction that each makes compared to the simple form. In particular, completive forms would seem to be more semantically marked—relative to simple forms—than stative forms. While completive inflection conveys some unexpected or unwanted, often negative implications, statives are more contextually and emotionally neutral. This could explain why children seemed to be relatively insensitive to the priming manipulation for statives (Study 1) as compared to completives (Study 2).

Before we end by drawing out a number of theoretical implications of the present findings, it is important to consider the extent to which the findings generalize to children's everyday language use. In terms of the *absolute* rates of simple versus complex verb forms produced, it is clear that children's behavior in the experiment is very different from their behavior in more everyday contexts. Across both studies, the vast majority of verbs were produced in complex form at a rate of between 50% and 80%, as opposed to around 10% in everyday language use. This is almost certainly due to our priming of complex forms—a conclusion supported by the fact that, at least for Study 2, the rate of complex‐form production increased over the course of the study. Presumably, had we not primed complex forms, children would have produced them at a rate of 10%, or—given that the picture contexts gave no particular reason to use complex over simple forms—even lower.

However, in terms of the *relative* (rather than absolute) rates of simple versus complex form production across verbs, the finding of a correlation between children's production data and distributional corpus data (albeit from adult speakers) is in itself evidence that the findings of this study generalize to everyday spoken Japanese. We can see no plausible mechanism by which boosting the usage of complex forms either (a) overall or (b) at an increasing rate as priming built up over the course of the study could yield the observed by‐verb pattern (given that trials were always presented in random order).

Given that the priming methodology almost certainly considerably boosted the use of complex forms, the present results do not constitute evidence that frequency *trumps* morphophonological or semantic complexity. Presumably even the verbs with the highest proportion of complex uses would have been produced overwhelmingly in simple form, had we not primed complex forms.^5^ What the results do show is that if we *control for* morphophological complexity—by effectively asking children to produce a complex form on every trial, an effect of relative input frequency is still observed.

The present study therefore provides perhaps the clearest evidence to date for an effect of input frequency on children's acquisition of inflectional morphology. Most—indeed, perhaps all—previous studies that have found apparent effects of input frequency in this domain have failed to control for morphophonological complexity (i.e., for the fact that high‐frequency forms tend to be shorter and simpler). By focusing on a system that shows by‐verb variation in the relative frequency of simple and complex forms, the present study has allowed us to investigate, and demonstrate, an effect of input frequency, controlling for morphophonological complexity.

From a broader language‐acquisition perspective, these types of frequency effects (see Ambridge et al., 2015, for a review) are clearly most straightforwardly compatible with input‐based constructivist accounts, under which systems of inflectional morphology—and, indeed, all linguistic subsystems—are built gradually on the basis of the input. They are not necessarily incompatible per se with accounts that assume innate knowledge of formal categories, principles or parameters (see, e.g., Yang, 2004). They do, however, present a considerable challenge in that such accounts need to incorporate a role for input‐based learning and at the same time explain exactly what role in the learning process is being played by innate formal knowledge (whose inclusion has traditionally been motivated by the presumed insufficiency of input‐based learning).

From a broader cognitive‐science perspective, these findings contribute to a growing body of evidence which suggests that effects of input frequency are ubiquitous in almost every domain of human and animal learning and behavior, including music (e.g., Temperley, 2007), mate choice (Grammer & Thornhill, 1994), and solving logic puzzles (including, for pigeons, the famous Monty‐Hall problem; Herbranson & Schroeder, 2010). They also add to a growing consensus that what is important in learning is not raw frequency (after all, in absolute terms, most of the complex forms elicited in the present study are extremely rare), but contingency: the link between a “predictor” (here, the verb) and a particular “outcome” (here a simple vs. a completive form), relative to the background rates of those occurrences (here, overall rates of simple vs. completive forms in the corpus, collapsing across all verbs). In fact, the importance of contingency has long been appreciated in the animal learning literature (e.g., Rescorla & Wagner, 1972), it has only recently begun to make inroads into the domain of cognitive science and, in particular, language acquisition (e.g., Ramscar, Dye, & Klein, 2013; Ramscar, Sun, Hendrix, & Baayen, 2017).

In conclusion, the present research (particularly Study 2; completives) provides support for the prediction of an input‐based account that the relative accessibility or representational strength of simple and complex forms of the same verb is related to the relative frequency of these competing forms in the input. The challenge for future work is to situate these types of competition effects into an account of the acquisition of inflectional morphology that is consistent with what we know about both language acquisition and human learning in general.

## Notes

1 The stative form diachronically is a reduced form from the combination of connective *‐te* and subsidiary verb *i*‐ “be, exist” (cf. Kudo, 1995). Therefore *tabe‐te‐ta* is reduced from a compound verb construction *tabe‐te i‐ta*, and the reduced form is common in spoken Japanese.

2 The completive form is also a reduced form, and the non‐reduced construction consists of connective ‐*te* and a subsidiary verb *shimaw*‐ “end.” The reduced form is very common in spoken language.

3 Another advantage of focusing on this system is that the agglutinative nature of Japanese verb inflection (verbs can be modified by attaching one or many morphemes) allows a clear definition of the simple and complex forms, compared to more fusional systems like Spanish person/number marking (e.g., ‐*a* (third‐person singular) is more prototypical than ‐*amos* (first‐person plural) in terms of phonology but not necessarily of morphology or of function).

4 To the extent that the non‐significant interaction is interpretable, the positive sign suggests that the effect of input bias—though itself non‐significant—showed a tendency to increase slightly over the course of the experiment.

5 Consequently, our results do not straightforwardly constitute evidence against accounts under which children proceed developmentally from simple forms to complex forms by the addition of morphemes (Iwatate, 1981; Takanashi, 2009).
